# Metabolic responses of a phototrophic sponge to sedimentation supports transitions to sponge-dominated reefs

**DOI:** 10.1038/s41598-017-03018-y

**Published:** 2017-06-02

**Authors:** Andrew Biggerstaff, David J. Smith, Jamaluddin Jompa, James J. Bell

**Affiliations:** 1 School of Biological Sciences, Victoria University of Wellington, Wellington, 6140 New Zealand; 2 Coral reef Research Unit, School of Biological Sciences, University of Essex, Essex, CO4 CSQ United Kingdom; 3 Research and Development Centre on Marine, Coastal and Small Islands, Hasanuddin University, Km. 10, Makassar, Indonesia

## Abstract

Declines in coral abundance have been linked to increased sedimentation at many locations across the world and at some of these locations there have been subsequent increases in sponge abundance. These shifts appear counterintuitive as sponges are suspension feeders and many rely on photosymbionts for carbon. At a sedimented reef in Indonesia (Wakatobi) corals have declined and the photoautotrophic sponge *Lamellodysidea herbacea* is now abundant. We hypothesise that this is partly due to *L. herbacea’s* ability to clear its tissues of high levels of settled-sediment and compensate for associated metabolic demands by altering its respiration rate. Negligible detrimental effects to sponge tissue were observed after treatments up to five times the sedimentation rate of the highly sedimented reef. Rapid sediment clearance occurred that was potentially aided by mucus production. Finally, high sediment exposure caused an immediate reduction in respiration rate, likely due to reduced pumping to prevent clogging, whereas sustained high sedimentation caused an increase in respiration rate, potentially due to the energetic cost of mucus production. Our study provides evidence that some sponges can tolerate environments that appear unsuitable to many corals and with increased sedimentation this acclimation may support further transitions to sponge dominated reefs in the future.

## Introduction

Coral abundance is declining on most of the world’s coral reefs despite efforts to try and reverse these trends^1^. These declines have been attributed to local anthropogenic stressors, such as overfishing of reef systems^2, 3^, destructive fishing practices, direct coral removal and increased sediment deposition, as well as global-scale factors, such as ocean acidification, and both chronic and acute (El Niño Southern Oscillation) increases in sea surface temperatures^4, 5^. Following declines in the abundance of reef building corals other benthic organisms, such as macroalgae, soft corals, corallimorpharians, and sponges, have been reported to increase in abundance creating shifts in benthic dominance^6^. These increases can be attributed to the new environmental conditions being more suited to the new suite of species, or the remaining species being either more phenotypically plastic or tolerant to the environmental changes than scleractinian corals.

Sponges fulfil many functional roles within reef ecosystems^7^ and therefore changes in their abundance may have important ecosystem consequences. In multiple tropical locations globally, declines in coral abundance have been followed by substantial increases in sponge abundance^8^. In contrast, mass mortality events of certain sponge species have also been observed over the past 30 years at both temperate^9^ and tropical^10^ locations, some of which have been attributed to disease outbreaks^11^. In tropical regions coral declines as a result of increased sedimentation rates have often been attributed to the sediment impairing coral symbiont photosynthesis, however several sponge species that have become dominant after sediment induced coral to sponge transitions also contain photosymbionts^12–16^. This suggests there is some differential ability between sponges and corals to tolerate sediment, and that sponges may have acclimation mechanisms or responses to enable survival in sedimented environments.

Sedimentation rates on coral reefs have increased in many geographic regions as a result of terrestrial land clearing and coastal construction^17^. Sedimentation can act as a physical stressor for many benthic reef organisms with the potential to smoother them, inhibit photosynthesis, block filtration apparatus and impair reproductive success, with corals often being particularly affected^18^. Sediment has also been identified as a stressor for sponges and can cause tissue abrasion, reduced post-larval survival, clogging of the aquiferous system during pumping and loss of photosymbionts^19^. Suspended sediment may negatively affect phototrophic sponges as a result of turbidity reducing the light available to symbionts and potentially clogging the aquiferous system. Burial by settled sediment can be fatal for phototrophic sponges as no light reaches the holobiont^20^. However, some heterotrophic sponges can survive even when completely buried in sediment^21, 22^. Sponges have a number of passive morphological sediment clearance adaptations, such as growth form^23^ and spicule projections^24^. They also have active physiological responses, including ‘backwashing’^25^, oscular contraction, cessation of pumping, and mucus production^26^.

Exposure to sediment is not exclusively detrimental for all sponge species as some sponge species actively incorporate sediment particles into their skeletal tissues and experimental application of sediment has even resulted in increased growth rates of their skeletal tissues^27^. This active selection of sediment particles appears to differ between sponge habitat, with soft sediment sponges selecting particles based on size, and sponges from hard benthic environments appearing to also select based on sediment mineralogy^28^. For example, the active selection and incorporation of crystalline silica by *Chondrosia reniformis* has been shown to increase the rate of collagen production, which form spongin fibres, a key component of the skeletal framework^29^. Furthermore, it has been hypothesised that the incorporation of sediments in the skeletal framework can save energy expenditure on spicule production and reinforce surface tissues to increase protection against spongivory^30^. However, the importance of sediment clearance mechanisms to reduce the negative effects associated with non-incorporated or excess sediment smothering sponges is often discussed in tandem with sponge sediment incorporation^27, 30^.

To the best of our knowledge, no studies have calculated the metabolic demand resulting from sponge mucus production. However, studies on coral mucus production as a sediment clearance mechanism have shown increased carbon requirements when mucus is produced^31^. Active responses to increased sedimentation by sponges are therefore likely to change rates of energy expenditure: responses such as mucus production will increase energy requirements, while responses such as cessation of pumping will decrease energy requirements. Four published studies of heterotrophic sponges have examined respiration rates in response to experimentally manipulated sedimentation rates, with three focusing on suspended sediment^32–34^ and one on settled sediment^35^. However, we are not aware of any studies of phototrophic sponge species and existing studies of heterotrophic sponges were conducted with sponges from locations where sediment has not induced coral to sponge dominance transitions.

**Figure 1:**
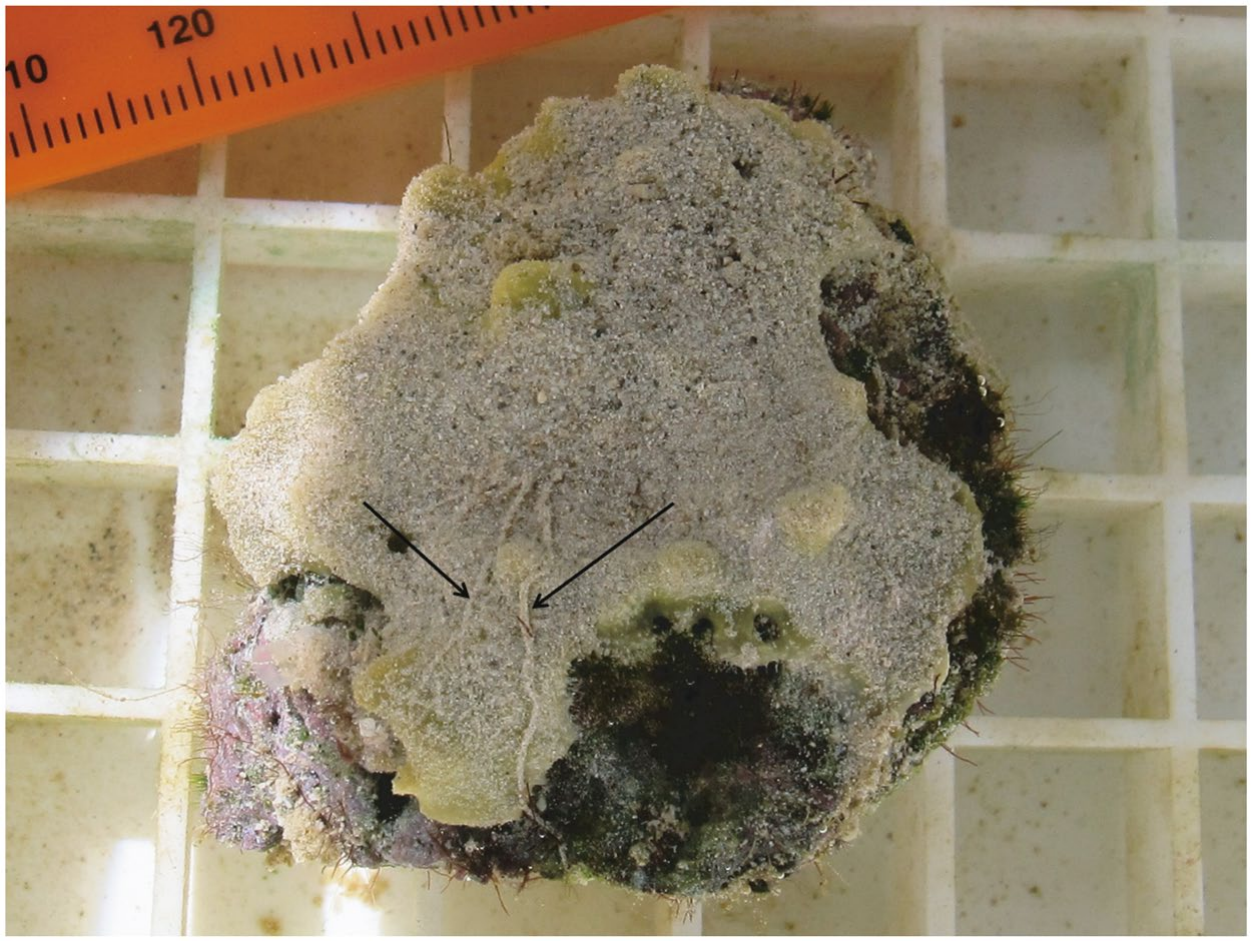
*L. herbacea* 24 hours after the x 5 sediment treatment that shows visible mucus trails marked with arrows.

*Lamellodysidea herbacea* (Fig. 1) is the most abundant sponge within the Wakatobi Marine Biosphere Reserve (WMBR), UNESCO, Indonesia representing over 40% of the total sponge abundance^14^. This species is also highly abundant on a sedimented reef system, Sampela^14^, where increases in sponge density^14, 36^ and decreases in coral cover^14, 37^ have been observed over the past decade. *L. herbacea* has an encrusting growth form, lacks spicules and has an irregular skeletal framework of spongin fibres (~50–150 µm width) all of which are cored with incorporated sand and sediment particles^38^. *L. herbacea* supports a dense population of cyanobacterial photosymbionts (*Oscillatoria spongeliae*), which represent approximately 50% of the volume of cells within the holobiont^39^. The effect of turbidity and sedimentation on the energetics of corals, which have zooxanthellae as photosymbionts, has been widely studied for a range of species, with high levels of turbidity and sedimentation often heavily restricting the range of their realised niche^40^. Comparatively, when sponge species contain photosymbionts they are predominantly cyanobacterial, and there have been few studies that have assessed the effects of turbidity and sedimentation on cyanobacterial sponge symbionts. However, a recent study used Pulse Amplitude Modulated (PAM) fluorometry and measurements of light intensity in the WMBR to show that the cyanobacterial photosymbionts of *L. herbacea* were photoacclimated to different *in situ* light levels (Sampela: low light, Pak Kasims high light) as a result of different environmental turbidity. In addition, extreme tissue discolouration and necrosis in shading experiments that ran for 14 days revealed that *L. herbacea* was reliant on its symbionts for survival^16^. Furthermore, this study showed that these symbionts could rapidly photoacclimate after shock transitions from high light to low light environments during transplantations. These results show how the phototrophic *L. herbacea* was able to cope with the higher turbidity caused by greater suspended sediment potentially helping to explain the coral to sponge transition that has been observed at this highly sedimented reef system. However, earlier studies noted that *L. herbacea* tissue is almost always clear of settled sediment even in areas where a large amount of sediment is falling out of suspension^14^. This suggests *L. herbacea* has a currently unknown mechanism for removing settled sediment from their tissue surface. Therefore, to fully explain how *L. herbacea* is surviving in these highly sedimented environments it is necessary to determine the mechanisms by which *L. herbacea* copes with increased levels of sediment settling on its tissue. This will be essential for predicting the fate of *L. herbacea* if sedimented reefs become more abundant in the WMBR region and elsewhere in the Indo-Pacific where this species occurs.

We performed laboratory-based additions of different levels of settled sediment to *L. herbacea* originating from a low sediment environment. These were used to: define the effect of different levels of sediment deposition on sediment clearance rates; determine the immediate and sustained effects of these sediment additions on the respiration rate; and to define the upper sediment accumulation tolerance levels before any visible detrimental effects occurred. We performed identical clearance rate experiments *in situ* at the low sediment site to assess the validity of laboratory trials. Finally, through a combination of the laboratory and *in situ* observations this study aimed to define the method of settled sediment clearance used by *L. herbacea*. From previous observations of *L. herbacea* in highly sedimented environments lacking sediment on their tissue surfaces, we predict that *L. herbacea* will cope with experimental applications of high sediment levels by initiating sediment clearance mechanisms. Furthermore, we hypothesise that the high abundance of this sponge relative to other photoautotrophs could potentially be a result of its ability to clear its tissues of high levels of settled sediment and compensate for associated metabolic demands by altering their respiration rate. Our study explores how effective sediment tolerance mechanisms in conjunction with high levels of sediment deposition could potentially shift the competitive abilities of benthic reef organisms resulting in regime shifts, with these results being applicable to multiple documented shifts on reef systems globally.

## Materials and Methods

### Study sites

This study was conducted at two reef systems within the WMBR: Sampela (05°29.6S, 123°45.26E) and Pak Kasims (05°28.00S, 123°45.25E) that are separated by a channel of water and a distance of approximately 1.5 km. The Sampela reef is situated off the coast of Kaledupa Island and the sedimentation rate in 2005 was measured at 2.02 (±0.17) mg cm^−2^ d^−1^, approximately four times that of reef sites in the surrounding area^41^. This high sedimentation rate has been attributed to the degradation of coastal ecosystems which trap terrestrial sediment, primarily mangroves and seagrass beds, by local inhabitants of both Kaledupa Island and also the stilted Sampela village, which is located on top of the reef flat at the Sampela reef^41^. Mangrove has been cleared for fire wood and extensive damage to seagrass beds has occurred as a result of food gathering activities. This effect is compounded by the topography of the Sampela reef system^14^, which forms a basin protected on the North, West and South by reef walls, trapping sediment that is washed into the basin, reducing the flushing rate and allowing for resuspension of trapped sediment. Sampela has been defined as a sponge-dominated reef and a reduction in coral cover has occurred over the last decade from over 30%^36^ cover to less than 10%^14^; at the same time there has been a 50% increase in sponge density^14, 37^. In addition, a significantly reduced rate of coral recruitment at Sampela has been reported compared to adjacent less sedimented reef sites^42^. The composition of the sediment settling on the Sampela reef has been defined as predominantly carbonate based with a minimal clay fraction (<7%)^43^. Direct comparisons of clay and carbonate based suspended sediments of a similar grain size applied at a similar concentration have shown that clay sediments increase the respiration rate of sponges by 35% whereas carbonate increase the respiration rate by only 12%^32^. This indicates that minerology of clay based sediments can significantly alter the metabolic rate of sponges, with clay based sediments having been shown to impact sponge respiration rates on multiple occasions^23, 34^, cause reductions in pumping^44^ and even mortality^20^. The Pak Kasims reef has a much lower rate of sedimentation, with the sedimentation rate measured as 0.76 (±0.08) mg cm^−2^ d^−1^ in 2005^41^. The Pak Kasims reef system has no large human population nearby and forms a large reef wall^14^ that provides a greater potential for flushing of sediment than the Sampela reef system basin. Despite the differences in sedimentation rates, other environmental variables such as sediment mineralogy^43^, mean current speed^14^, salinity^45^, pH^46^ and temperature^14^, show little variation between sites within the WMBR. In contrast to the Sampela reef system, Pak Kasims is a coral-dominated environment with a mean coral cover of 35% and half the total sponge density of Sampela^47^. *L. herbacea* abundance is also much higher on the Sampela reef system (110 individuals m^−2^) than the Pak Kasims reef system (20 individuals m^−2^)^14^.

### *L. herbacea* collection and aquaria facilities

The anatomical species identification of *L. herbacea* was confirmed using DNA barcoding. Amplified and sequenced Internal Transcribed Spacer 2 (ITS2) regions of reference *L. herbacea* specimens collected from the WMBR were included in a phylogeny with existing published ITS2 sequences for *L. herbacea* ^48^ and using *Dysidea arenaria* sequences as an outgroup^48^, with all WMBR ITS2 sequences nesting within the *L. herbacea* clade. *L. herbacea* were collected from the Pak Kasims reef flats (approx. depth 2 m ± 1 m) as this study was designed to ascertain the effects of increased sedimentation on *L. herbacea* originating from a coral-dominated environment with a low sedimentation rate. In addition, with the larval dispersal of *L. herbacea*, the close proximity of the Pak Kasims and Sampela reefs and no observable barrier to connectivity between the *L. herbacea* from these reefs are highly unlikely to be genetically divergent. Therefore, results of experiments performed on Pak Kasims *L. herbacea* are likely to be representative of Sampela *L. herbacea*. Furthermore, with observations suggesting that this sediment induced regime shift has occurred over the past one or two decades this would suggest that major site specific genotypic adaptations of *L. herbacea* to sediment at Sampela would be unlikely in such a short evolutionary time frame. All sponges collected had no tissue necrosis and were 20 mm–50 mm wide. *L. herbacea* were removed undamaged and attached to a piece of unbroken underlying substratum. In the aquaria, non-*L. herbacea* encrusting organisms and infaunal organisms were removed from the attached substrate. *L. herbacea* were then allowed to adjust to aquaria conditions for one week before experiments began.

A flow through system provided unfiltered seawater from the coastal region of Pak Kasims to the base of aquaria with a mean flow rate of 0.41 (±0.04) cm^3^ s^−1^ creating an effective 100% water change approximately every 1 hour 20 minutes. Each aquarium had an airstone positioned with the water inflow tube and the overflow in the opposite corner of the aquarium to the *L. herbacea* samples to create a low flow environment. This low flow environment was created to remove any contribution to sediment clearance from aquarium water flow to result in the measurement of active sediment removal mechanisms by *L. herbacea*. A 12:12 hr light:dark cycle was applied from a sunlight strip bulb (AquaOne, T8) and the water temperature was maintained between 26.9–28.1 °C. *L. herbacea* not in experimental groups were maintained in these conditions for over 8 weeks without any, necrosis, tissue discolouration or observable reductions in size, indicating that these conditions are suitable for *L. herbacea*.

### Preliminary assessment of Sampela sediment deposition rates and grain size

Sediment traps were used to assess the level of sediment deposition at the Sampela reef. Analysis of this data resulted in the following sediment treatments scaled to the rate of sediment deposition at Sampela: x 0.5 (2.5 mg cm^−2^ d^−1^), x 1 (5 mg cm^−2^ d^−1^), x 2 (10 mg cm^−2^ d^−1^) and x 5 (25 mg cm^−2^ d^−1^). These treatments were made of equal parts: 125–250, 63–125 and 38–63 µm fractions with sediment collected from the Sampela reef. For full: sediment trap experimental design, protocol for sediment processing, results and selection of treatment levels see Supplementary Methods.

### Sediment clearance rates

The sediment treatments for clearance experiments were x 0.5, x 1, x 2 and x 5; controls had no sediment addition. Results from preliminary analyses indicated that the surface area of *L. herbacea* was strongly positively correlated with dry weight (R^2^ = 0.855, F_(1,18)_ = 106.371, p < 0.001). Therefore, sediment added was scaled to the treatment group and surface area of the *L. herbacea* and was suspended in seawater and evenly pipetted over the tissue surface (seawater pipetted over surface for controls). The experiment was conducted in five aquaria with one *L. herbacea* from each group in each tank. Photographs were taken of each *L. herbacea* with an adjacent scale bar immediately before the addition of sediment, immediately after the addition of sediment, 4 hours after the addition of sediment, and then each day, 1–7 days after the initial sediment had been added. These photographs were then analysed using Image-J to calculate the percentage of sponge covered by sediment. In addition to the single sediment addition experiment, a laboratory-based sediment tolerance experiment was performed where the x 5 sediment treatment was applied to sponges each day. Five *L. herbacea* were housed in separate aquaria and the same method was used for sediment addition. *L. herbacea* were photographed with an adjacent scale bar immediately before and after each sediment addition each day for 5 days until tissue discolouration was observed.

*In situ* clearance experiments were performed to ascertain whether the laboratory experiments were representative of field results. The *in situ* clearance experiment was performed at Pak Kasims to ensure sponges came from the same location as those in laboratory experiments. The experiment was performed at Pak Kasims, as opposed to being transplanted to Sampela, as this allowed for the same pulse addition of sediment as in the laboratory and also so the x 0.5 treatment could be applied. At Pak Kasims *L. herbacea* were selected using the same selection criteria as for the laboratory experiments, with a minimum of 5 m horizontal distance between sponges and allocated to random treatment groups. The *in situ* clearance experiment sampling and sediment addition methodology was identical to the laboratory experiments with the exception of the 4 hour and 5–7 day sampling not being completed *in situ*. The controls throughout the *in situ* clearance experiment represented background settled sedimentation minus any sediment clearance.

### Respiration measurements

All respiration measurements were performed in cylindrical respiration chambers (40 mm diameter and 55 mm deep) using an optical oxygen meter (PreSens, Fibox 3). For detailed descriptions of respiration measurements including; calibrations, dark adaptations, determining recording time and methods of standardising for volume and weight of *L. herbacea* samples see Supplementary Methods.

### Immediate effect of sediment addition on respiration

*L. herbacea* were randomly allocated to control, x 0.5, x 1 and x 2 sediment treatment groups (n = 10). The respiration rate for each of these *L. herbacea* was then measured for 15 minutes. After 15 minutes the temperature probe was removed and the sediment treatment was added. The sediment could not be carefully added to the surface of the *L. herbacea* tissue as in the clearance experiment, due to the blacked out chambers for dark adaptation, so the amount of sediment was calculated for the entire base of the chamber and added through the hole in the lid of the chamber for the temperature probe. This when trailed resulted in an even spread of the sediment across the base of the chamber and therefore also the *L. herbacea*. The temperature probe was then replaced and the respiration rate measured for a further 15 minutes.

### Effect of sustained sediment addition on respiration

*L. herbacea* were housed individually in aquaria and were randomly allocated to control, x 0.5, x 1 and x 2 sediment treatment groups (n = 7). The respiration rate for each *L. herbacea* was measured for 30 minutes before the first sediment treatment was added, with this measurement representing day 0. *L. herbacea* were then returned to their aquaria and the first sediment treatment was added by pipetting the sediment on to the surface of the *L. herbacea* as described for the sediment clearance experiments. Following the application of the sediment treatment *L. herbacea* were then left with the settled sediment treatment in place for 24 hours in aquaria before measuring the respiration rate again, with this measurement representing day 1. This 24 hour spacing was an attempt to allow any effects attributed to the immediacy of the sediment addition to pass, allowing measurement of the longer term effects of sustained sediment addition on respiration rate. This daily cycle of respiration measurements and then subsequent addition of the next sediment treatment was carried out over 5 successive days.

### Data Analysis

All statistical analyses were performed using SPSS (version 20). Data transformations conducted to meet the assumptions of statistical tests have been stated. Levene’s and Shapiro-Wilk tests were used to test for homogeneity of variance and normality respectively. Due to non-linear sediment clearance data over time, Log_10_ transformations were performed to ensure that sediment clearance data for all sediment treatments had linear regressions with time that had an R^2^ > 0.80 for inclusion in General Linear Models (GLMs). For all statistical analyses where multiple measurements were recorded for the same *L. herbacea* over time, a random factor of individual nested within sediment treatment was included in the model. For the comparison of *in situ* vs. laboratory sediment clearance this term was then further nested within ‘Experiment’.

GLMs were performed for both the laboratory and *in situ* sediment clearance experiments. Response variable: percentage cover of sediment; Dependent variables: sediment treatment (fixed factor) and time (covariate). For all laboratory clearance statistical analyses the control percentage cover values were removed as these were all 0. The same GLM was also applied to the combined experimental data set with the additional dependent variable of experiment (fixed factor). For this comparison of laboratory and *in situ* experiments all of the temporal sampling points and groups that were not in both experiments were removed (controls and laboratory 4 hr and day 5–7 data). Furthermore, a two way ANOVA was performed to determine if there were any significant differences in day 0 sediment percentage cover between experiments; Response variable: day 0 sediment percentage cover, Dependent variables: sediment treatment (fixed factor) and experiment (fixed factor).

For the immediate effect of sediment addition on the respiration rate experiment, a two way Analysis of Variance (ANOVA) was performed. Response variable: respiration rate; Dependent variables: before/after sediment addition (fixed factor), sediment treatment (fixed factor), and interaction between these two factors. An ANCOVA was also performed to define any significant differences between sediment treatments in post sediment treatment respiration rates normalised to pre-sediment treatment respiration rates. Response variable: respiration rate post treatment; Dependent variables: respiration rate before sediment treatment (covariate), and sediment treatment (fixed factor). Finally, a one way ANOVA was performed to determine if there were any significant differences between sediment treatments in pre-sediment treatment respiration rates; Response variable: respiration rate before sediment treatment, Dependent variable: sediment treatment (fixed factor).

For the sustained sediment addition experiment a GLM was performed; Response variable: respiration rate; Dependent variables: sediment treatment (fixed factor) and time (covariate).

## Results

### Sediment clearance rates

Of the 123 *L. herbacea* used across all experiments, we only observed the full necrosis of one and the partial necrosis of another *L. herbacea* in the control group of the laboratory clearance experiment. Due to both these sponges being in the control group this was not considered to be linked to the sediment treatments. In addition, a single fragmentation event was seen in the clearance experiment of a sponge in the x 0.5 treatment. Between 1–2 days after sediment addition, a thin line of tissue necrosis (approx. 0.04 cm^2^) occurred where the tissue was indented allowing sediment to accumulate, resulting in two distinct *L. herbacea*, which both showed no sign of necrosis for the rest of the experiment. In addition, a potential method of active sediment clearance in the form of mucus production was observed as visible trails of clumped sediment in every *L. herbacea* in the x 1, x 2 and x 5 treatment groups for both the *in situ* and laboratory clearance experiments (Fig. 1).

**Figure 2:**
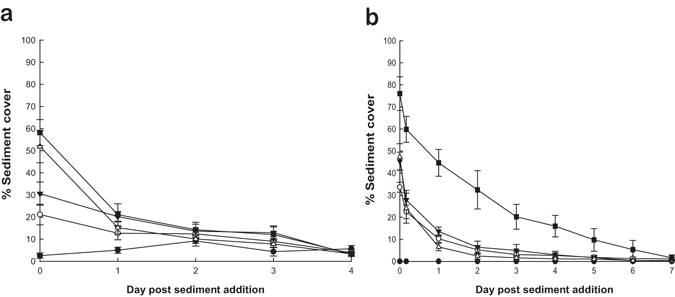
Changes in mean percentage sediment cover (±SE) on *L. herbacea* over time after sediment addition for all sediment treatment groups: control (filled circles), x 0.5 (open circles), x 1 (filled triangles), x 2 (open triangles) and x 5 (filled squares) for both *in situ* (**a**) and laboratory (**b**) experiments.

For the *in situ* clearance experiment the percentage cover of sediment significantly decreased with time (Table 1 and Fig. 2a). Significant differences were seen between sediment treatment groups with *post hoc* testing indicating that, the control group had a lower percentage sediment cover across the experimental period than the other treatment groups. However, no significant differences were seen between the sediment clearance patterns for the other sediment treatments. The laboratory clearance experiment showed a similar significant reduction in percentage cover of sediment with time (Table 1 and Fig. 2b) and there was also a significant difference between the sediment treatments (Table 1). *Post hoc* testing between sediment treatments indicated that the x 5 treatment group had a lower rate of sediment clearance than the other treatments, indicated by the shallower gradient of decreasing sediment (Fig. 2b), and that the x 0.5, x 1 and x 2 treatments in the laboratory were not significantly different. No significant difference was seen in the sediment clearance patterns between the laboratory and *in situ* clearance experiments (Table 1). However, this result needs to be considered in the context of the loss of power resulting from the removal of data points that could not be compared between both experiments (controls and laboratory data after 4 hrs and days 5–7 data). In addition, day 0 values for the *in situ* experiment did appear slightly lower than in the laboratory experiment (Fig. 2), however, these differences were not significant (square root transformed ANOVA_Experiment_: F_(1,40)_ = 2.404, p = 0.129). Despite these points, the clearance patterns, where most clearance occurred in the first 24 hours and then this rate slowed, were similar between the laboratory and *in situ* experiments for the x 0.5, x 1 and x 2 treatments (Fig. 2a). As there were no significant differences in sediment clearance patterns for x 0.5, x 1 and x 2 treatments in both the laboratory and *in situ*, this could indicate that variation in sediment application within this range has no significant impact on rate of sediment clearance (Fig. 2b).

**Table 1:** Summary table of the statistical models used in analysis of the laboratory and *in situ* clearance experiments and a comparison between them.

Experiment	Dependent variable	d.f.	F	p
Laboratory	Sediment treatment	3, 16	8.003	0.002
	Time	1, 159	545.065	<0.001
*In situ*	Sediment treatment	4, 20	13.613	<0.001
	Time	1, 99	52.150	<0.001
Laboratory vs. *In situ*	Sediment treatment	3, 35	7.422	0.001
	Time	1, 159	419.268	<0.001
	Experiment	1, 35	2.735	0.107

Response variable: percentage sediment cover (Log_10_ transformed), the dependent variables: sediment treatment (fixed factor), time (covariate) and *in situ* or laboratory (fixed factor).

As the x 5 treatment showed different clearance patterns between the laboratory and *in situ* experiments and the majority of the sediment was not cleared after 24 hours, the x 5 treatment group was not included in further laboratory respiration experiments. However, the tolerance experiment was performed and all *L. herbacea* were seen to survive daily additions of x 5 sediment treatment for five successive days with no necrosis and only one *L. herbacea* showed some tissue discolouration on the fifth day.

### Immediate effect of sediment addition on respiration

**Figure 3:**
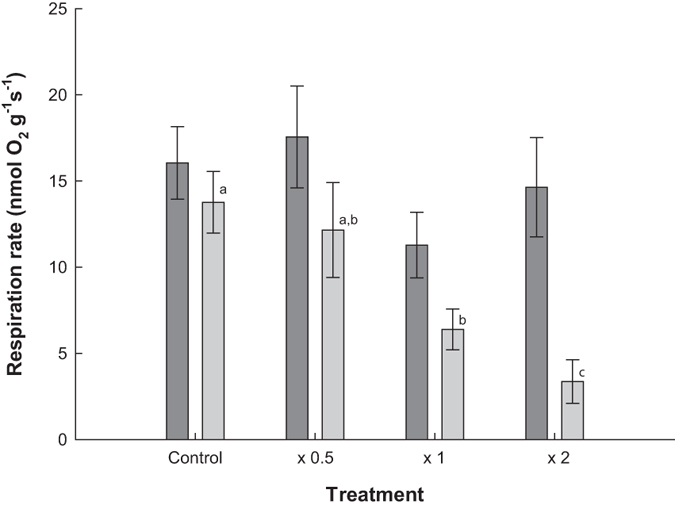
Mean respiration rates (±SE) of *L. herbacea* before (dark grey) and after (light grey) sediment addition for sediment treatment groups, with significant differences in after sediment respiration values normalised to before sediment respiration values (**a**,**b**,**c**).

There was no significant difference in the respiration rates between sediment treatments before the addition of sediment (sqrt trans. ANOVA_Treatment_: F_(3,36)_ = 1.009, p = 0.400). However, there was a significant interaction between Before/After sediment application and sediment treatment (Table 2). This indicated that different treatments showed significantly different responses in respiration rate to their level of sediment addition. *Post hoc* testing in the form of paired t-tests revealed that both the x 1 and x 2 sediment treatments showed a significant reduction in respiration rate post treatment, with the control and x 0.5 treatments showing no significant difference (Fig. 3). Significant differences were also observed between sediment treatments for respiration rates post sediment addition, normalised for respiration rates before sediment addition (Table 2), with *post hoc* results labelled on Fig. 3.

**Table 2:** Summary table of the immediate effect of sediment addition on respiration data: two way ANOVA, response variable: respiration rate (square root transformed), the dependent variables: Before/After treatment (fixed factor) and sediment treatment (fixed factor) and an interaction, and the ANCOVA, response variable: respiration rate after treatment application (square root transformed), the dependent variables respiration rates before treatment application (covariate) and sediment treatment (fixed factor).

Statistical test	Dependent variable	d.f.	F	p
Two way ANOVA	Before/After	1, 36	26.910	<0.001
	Sediment treatment	3, 36	5.314	0.004
	Before/After*Sediment treatment	3, 36	4.470	0.009
ANCOVA	Before treatment respiration	1, 35	3.623	0.065
	Sediment treatment	3, 35	9.621	<0.001

### Effect of sustained sediment addition on respiration

**Figure 4:**
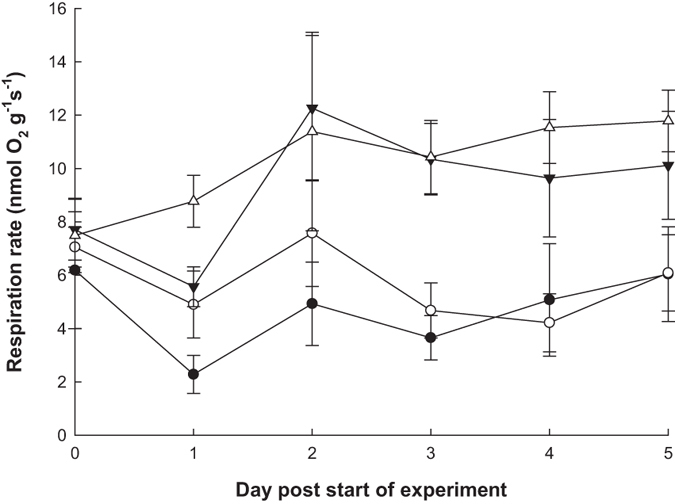
Changes in mean respiration rates (±SE) of *L. herbacea* over time to examine the effect of sustained exposure to settled sediment for sediment treatment groups: control (filled circles) x 0.5 (open circles), x 1 (filled triangles) and x 2 (open triangles).

The respiration rate for the x 1 and x 2 sediment treatment levels significantly increased with time during the experiment (Table 3 and Fig. 4). In contrast, the control and the x 0.5 sediment treatments respiration rate showed no significant change with time (Table 3) indicating that these treatments had no effect on respiration rate during the experiment. A significant difference was found in the mean respiration rates across sediment treatments in the sustained sediment experiment (Table 3). *Post hoc* testing between sediment treatments revealed that respiration rates for the x 1 and x 2 sediment treatment groups were significantly higher than for the control and x 0.5 treatment groups (Fig. 4).

**Table 3:** Summary table of the statistical models used in analysis of all sediment treatments and data sets split into x 1 and x 2 treatment groups and control and x 0.5 treatment groups, for the sustained exposure to sediment respiration experiment.

Data set	Dependent variable	d.f.	F	p
All treatments	Sediment treatment	3, 24	12.670	<0.001
	Time	1, 139	2.758	0.099
x 1 and x 2	Sediment treatment	1, 12	0.591	0.457
	Time	1, 69	8.378	0.005
Controls and x 0.5	Sediment treatment	1, 12	2.154	0.168
	Time	1, 69	0.028	0.868

Response variable: respiration rate (square root transformed) the dependent variables: sediment treatment (fixed factor) and time (covariate).

## Discussion

Multiple reported shifts from coral to sponge-dominated systems on reefs have been attributed to an increase in sedimentation rate^12–16^. Through the application of settled sediment treatments to *L. herbacea* the present study aimed to determine any potential methods of sediment clearance and determine the sediment clearance rate. In addition, this study aimed to define the immediate effect and the effect of sustained sediment exposure on the respiration rate of *L. herbacea*. We found that *L. herbacea* can clear their tissue surface of very high levels of settled sediment and can survive daily additions at these levels. We also observed that *L. herbacea* appears to produce mucus, with this potentially acting as a clearance method in response to additions of x 1, x 2 and x 5 the natural sedimentation rate at Sampela. Furthermore, we found that with the application of the x 1 and x 2 levels of sediment the respiration rate of *L. herbacea* decreases in the short term, but increases with sustained exposure creating contrasting temporal metabolic strategies. These results provide a better understanding of the likely mechanisms supporting a phototrophic sponge to survive and dominate in highly sedimented reef environments, which are conditions that would are largely unsuitable for many coral species^49^.

The similarities between *in situ* and aquaria *L. herbacea* sediment clearance patterns validated the use of the aquarium experiments. Furthermore, as the aquaria were designed to be low flow environments, these results indicate that *L. herbacea* is not reliant on natural water movement to clear settled sediment but it can actively clear sediment from the surface of its tissue at the levels used in respiration experiments. Sampela is heavily sedimented compared to other local reefs in the WMBR^41^ and has a comparable sedimentation rate to many other reefs defined as being heavily sedimented globally. Therefore, by increasing this already high rate by 5 times, the slower clearance rate in aquaria compared to other treatment levels was not unexpected. However, the similar clearance rates between the x 0.5, x 1 and x 2 treatment groups could indicate a clearance response that does not vary when exposed to sediment levels within this range. To further highlight *L. herbacea*’s sediment tolerance, other published studies that have experimentally applied pulses of settled sediment to sponges at approximately the corresponding *in situ* daily deposition rates, have all shown large amounts of tissue necrosis, symbiont death or reductions in size of the sponges^20, 35, 50^.The origin of all *L. herbacea* used in our experiments were from the Pak Kasims reef system that has a low sedimentation rate, meaning our results cannot be attributed to long term acclimation to high levels of sediment.

We found that mucus is a potentially important sediment clearance mechanism with mucus being produced by all sponges in the x 1, x 2 and x 5 treatment groups in both *in situ* and laboratory experiments. This is the first recording of *L. herbacea* producing mucus and there have only been a few papers that have documented sponge mucus production as a sediment clearance mechanism^26, 32, 51, 52^. However, due to the broad geographical and taxonomic range of sponge species exhibiting mucus production as a clearance mechanism, the low number of reports is probably a result of a paucity of observations as opposed to mucus production being a rare trait. The high energetic cost of mucus has been well studied in corals, which combined with the reduced photosynthetic capacity caused by high sedimentation rates is often used to explain coral declines in highly sedimented areas^31^. However, this does not seem to be a problem for *L. herbacea* as this study has shown an ability for this species to up regulate its respiration rate in response to high levels of sustained sediment addition, in conjunction with observed mucus production and no observable detrimental effects over a 5 day period.

The metabolic rate of sponges being phenotypically plastic in response to environmental cues and the discussion of this as an acclamatory response has been reported previously^53^. The 50 and 80% reduction in the mean respiration rate immediately after sediment addition for the x 1 and x 2 treatment groups, respectively, are most likely due to a reduction or cessation of pumping as a short term response to prevent clogging of the aquiferous system. Sponges are obligate filter feeders but have little selective control over the size of particles entering the aquiferous system^54^. A reduction or the complete arrest of pumping activity in sponges has been observed in response to both natural sedimentation increases^55^ and experimental sediment applications^26, 56^. This immediate reduction in pumping activity and consequently respiration rate could represent a short term protective response, whereby the sponge does not immediately initiate any potentially energetically costly sediment clearance mechanisms, due to the possible transient nature of sedimentation. Whether this reduction in pumping activity is an active response by the sponge to prevent further clogging or a passive response due to clogging of the aquiferous system remains unclear. However, both scenarios result in less additional sediment entering the sponge, avoiding having to initiate active declogging mechanisms such as backwashing^25^.

The non-significant reduction in respiration rate for the x 0.5 treatment group immediately after sediment addition could indicate that there is a threshold sediment level that needs to be reached before there is a reduction in respiration rate, which falls between the x 0.5 and x 1 treatment levels. However, it is also possible that the effect size of the decrease in respiration rate after the sediment addition for the x 0.5 treatment group is too small for a significant response to be to be identified within this study. Furthermore, the significantly lower x 2 than x 1 sediment treatment post sediment respiration rate that had been normalised for before sediment respiration rate provides some indication that the reduction in sediment scales with sediment loading.

The significant increase in respiration rate by approximately 50% for both the x 1 and x 2 treatment groups during the sustained daily sediment addition experiment contrasted with the immediate effects of sediment addition. This contrasting metabolic response to settled sediment, with the immediate reduction in respiration rate following the application of sediment but then an increase in respiration rate with sustained sediment exposure has not previously been documented for sponges. The sediment treatment groups that increased in metabolic rate with sustained sediment addition were also the treatments where mucus was observed to be produced, with no mucus production observable in the lower sediment treatments. Furthermore, an earlier study reported visual evidence of mucus production by non-phototrophic sponges in an elevated suspended sediment treatment, with sponges in this treatment group also showing an increase in respiration rate^32^. During sediment treatments, the highest settled sediment level where mucus production was not observed was 2.5 mg cm^−2^ d^−1^ and the lowest level where mucus production was observed was 5 mg cm^−2^ d^−1^. The actual recorded settled sediment deposition rate for Sampela (4.40 (±0.30) mg cm^−2^ d^−1^ Supplementary Material) is very close to a settled sediment treatment where mucus was observed to be produced by *L. herbacea*. However, the length of our sustained exposure to sediment experiment (only 5 days) needs to be considered in context of the natural levels of sedimentation on the Sampela reef system, which we have identified as being chronic. Whether this increased respiration rate caused by sustained exposure to sediment can be maintained over longer temporal scales than the one used in this study has yet to be determined. In addition, longer experimental periods would need to be employed to ascertain if this increase in sediment has longer term impacts to *L. herbacea*. Histological evidence of mucus secreting cells of *L. herbacea* and quantitative measurements of mucus production in response to sediment exposure would assist in confirming the hypothesis that mucus production in *L. herbacea* is an important sediment clearance mechanism. Furthermore, these results would aid in confirming whether the level mucus production positively correlates with the respiration rate of *L. herbacea* during exposure to settled sediment treatments, which is highlighted as a potential relationship from the results produced in this study.

Even though there is a high chance of genotypic similarity between Pak Kasims and Sampela given their proximity and the experiments within this study focussing on traits that can change in response to environmental cues, there is still the potential for non-reversible phenotypic plasticity during early life-history stages in response to sedimentation at Sampela. However, such variable traits are generally associated with variations in body plans that could infer sediment tolerance such as morphology and spicule projections. There were no observable differences in the morphologies of *L. herbacea* between Pak Kasims and Sampela and *L. herbacea* lack spicules, however future experiments similar to those within this study conducted on *L. herbacea* originating from Sampela could ascertain whether any longer term phenotypically plastic traits to sediment adaptation are present at Sampela. Despite these points, our results strongly indicate that the increased respiration rate, and potentially mucus production, are responses by *L. herbacea* to high levels of sedimentation. Therefore, due to the dominance of *L. herbacea* at Sampela it seems likely that under these environmental conditions any increased respiration rate which may be associated with clearing its tissue of sediment could be outweighed by the benefit of *O. spongeliae* photosynthesis with clear tissues. *L. herbacea* has been previously shown to be heavily dependent on *O. spongeliae* with extreme shading producing large amounts of necrosis^16^. However, this earlier study also showed substantial declines in a photophysiological metric with is proportional to photosynthetic rate when sponges were transplanted from a high light to low light environment. This provides some evidence to support the sediment clearance costs potentially being outweighed by the benefits of photosynthesis with clear tissue, however future studies measuring photosynthesis to respiration rate ratio with clear tissue surfaces and during sediment clearance would confirm this.

The potential for the incorporation of sediment into the skeletal framework of *L. herbacea* during our experiment also needs to be considered with regard to the sustained sediment respiration results. *L. herbacea* are known to contain inorganic particulate matter as components of their skeletal framework that are of a size that overlaps the sediment fractions used in this experiment (~50–150 µm)^38^. In addition, a species within the same family, *Dysidea etheria*, has been shown to actively incorporate sediment deposited on their surface with this increasing the growth rate of skeletal fibres^27^, a process which could increase the metabolic demand. However, there was no significant increase in the metabolic rate for the sustained x 0.5 sediment treatment with this treatment level in excess of any potential sediment incorporation by *L. herbacea* during the sustained sediment exposure experiment. This was ascertained from sediment clearance results showing some sediment was still present after 24 hours and a new dose of sediment was applied every day. Therefore, if sediment incorporation was the cause of the increase in respiration seen in the sustained sediment experiment then an increase would have been expected in the x 0.5 treatment level. Despite this, future assessments of sediment minerology in the WMBR, any incorporation of these sediments by *L. herbacea* and any beneficial effects to *L. herbacea* growth rate could be utilised to test the hypothesis that *L. herbacea* is at least partially benefitting from exposure to sedimentation.

In conclusion, we have shown that a phototrophic sponge, which has come to dominate the benthic environment of a sedimented tropical reef, can survive very high levels of settled sediment by using different metabolic responses. With exposure to high levels of sediment the sponge immediately reduces its respiration rate, most likely due to the cessation of pumping as a short term protection mechanism, and then with sustained sediment exposure *L. herbacea* potentially initiates the observed mucus production to clear its tissues of settled sediment. Our results showing how *L. herbacea* responds to high levels of settled sediment supports an earlier study showing *L. herbacea*’s photosymbionts can acclimate to turbid low light environments^16^. to provide a likely explanation of how a predominantly phototrophic organism is able to proliferate at heavily sedimented sites. The results of this study provide a greater understanding of the mechanisms driving the transition of benthic dominance, which in this case appears to be caused by changes to sedimentation dynamics.

## Electronic supplementary material

Supplementary information
