# Supplementary material for: Metabolic responses of a phototrophic sponge to sedimentation supports transitions to sponge-dominated reefs
Source: Sci Rep. 2017 Jun 2;7:2725. doi: 10.1038/s41598-017-03018-y (PMC5457416; doi:10.1038/s41598-017-03018-y)
Supplement: Supplementary file 1 — Supplementary information [file 41598_2017_3018_MOESM1_ESM.pdf]

Metabolic responses of a phototrophic sponge to sedimentation supports transitions to sponge-dominated reefs

Andrew Biggerstaff, David J. Smith, Jamal Jompa and James J. Bell

Supplementary information

Supplementary methods: Preliminary assessment of Sampela sediment deposition rates and grain size

Cylindrical sediment traps (30 cm height, 50.26 cm^2 funnelled opening) were positioned at 5 m and 10 m below the reef crest (n = 5) at Sampela with a minimum horizontal distance of 20 m between traps. An aspect ratio of 3.75 was deemed sufficient as cylindrical sediment traps with an aspect ratio of ≥ 3 have been previously defined as efficient collectors at sites with a current velocity of up to 0.2 m/s [1], Samella's current velocity has been measured at 0.056 m/s [2]. Traps were collected after two weeks and this was replicated across 3, 2 week periods. Any visible organisms were removed and the remaining contents were wet sieved using fresh water through nested sieves creating sediment fractions of: >250 µm, 250-125 µm, 125-63 µm, 63-38 µm and <38 µm. All fractions were then dried at 80 °C to a constant weight.

Of the 30 sediment traps deployed five were unable to be recovered, three from 5 m and two from 10 m. The mean sediment deposition across all traps was 4.40 (± 0.30) mg cm^{-2} d^{-1}. Due to the potential for a minor loss of sediment reaching the *L. herbacea* tissue during application, but no potential for sediment gain, the decision was made to round this value up to the nearest mg. The following treatment levels scaled to the Sampela sedimentation rate were defined: x 0.5 (2.5 mg cm^{-2} d^{-1}), x 1 (5 mg cm^{-2} d^{-1}), x 2 (10 mg cm^{-2} d^{-1}) and x 5 (25 mg cm^{-2} d^{-1}). This range of treatments was chosen as it contains the mean environmental sedimentation rates for sites defined as highly sedimented within the last 5 years in: Bermuda[3], the Great Barrier Reef (GBR)[4], Tanzania[5], Tobago[6], Palau[7] and Palmyra Atoll[8]. In addition, our sedimentation range included the estimated mean threshold rate where excessive sediment induces coral recruit mortality on the GBR, 3 mg cm^{-2} d^{-1} [9]. The mean composition of the sediment fractions was: > 250 µm = 19.94 % (± 2.02), 125 - 250 µm = 23.02 % (± 0.83), 63 - 125 µm = 25.43 % (± 1.26), 38 - 63 µm = 20.32 % (± 1.63) and < 38 µm: =11.03 % (± 0.98). The majority of the > 250 µm

sediment fraction was comprised of the shells of mobile organisms, or large pieces of plant matter. Therefore this fraction was removed from the study as these components would likely be transient on *L. herbacea* tissue. The <38 µm was also removed due to a combination of its low percentage composition and because sediment traps are known to overestimate smaller grain size fractions that would permanently settle on to the substrate due to the removal by micro-currents that re-suspend these fractions[10]. As a result of the comparatively similar percentages of the remaining fractions, equal parts of the 125 - 250, 63 - 125 and 38 - 63 µm fractions were used for all sediment additions. Sediment for all experiments was collected from the Sampela reef system and processed through the nested sieves and dried in the same way as the sediment trap samples.

Supplementary methods: Respiration measurements

All respiration measurements were performed in cylindrical respiration chambers (40 mm diameter and 55 mm deep). The water was kept moving at a constant slow rate using a magnetic stir bar at the base, separated from the main chamber by a mesh disc. Oxygen and temperature probes from the optical oxygen meter (PreSens, Fibox 3) were inserted through the lid to take oxygen readings every second. Chambers were placed in a water bath of during the measurements to minimise temperature changes. Chambers were blacked out and each *L. herbacea* and the seawater were kept in the chamber with an airstone in the dark for 30 minutes prior to respiration measurements. This was to avoid photosynthesis by symbionts or any other organisms in the seawater altering the oxygen content. The oxygen meter was calibrated daily to 0 % oxygen using sodium sulphite and 100 % air saturation using an airstone, before taking measurements. Oxygen measurements from the meter were only recorded after initial fluctuations had stabilised. Respiration measurements were ended prematurely if the oxygen level fell below 70% to avoid any detrimental effects to the *L. herbacea*. From preliminary measurements 30 mins was determined as an optimal recording length to collect the largest amount of data without levels consistently dropping below 70%. The respiration rate of a piece of substratum with no *L. herbacea* attached was also determined at each sampling point. These were of approximately the same size, and harvested, cleaned and housed in the same way and at the same time as the pieces of substrata attached to the *L. herbacea*. This was to account for the approximate respiration rate of any missed organisms during the cleaning process and any temporal fluctuations in the organismal content of the unfiltered seawater. Fluid displacement was used to measure the volume of all *L. herbacea* samples and attached substratum to account

for differences in the volume of seawater. Once the experimental period had ended all *L. herbacea* were removed from their attached substratum and dried at 80 ^{o}C to a constant weight to standardise the respiration rate per gram of dry weight.

References

1. White, J. The use of sediment traps in high-energy environments in *Marine Geological Surveying and Sampling* (eds Haliwood E. A. & Kidd R. B.) 145-152 (Springer Netherlands, 1990).

2. Powell, A. L. et al. Reduced Diversity and High Sponge Abundance on a Sedimented Indo-Pacific Reef System: Implications for Future Changes in Environmental Quality. *PLOS ONE* **9**, e85253 (2014).

3. Jones, R. J. Environmental effects of the cruise tourism boom: sediment resuspension from cruise ships and the possible effects of increased turbidity and sediment deposition on corals (Bermuda). *B. Mar. Sci.* **87**, 659-679 (2011).

4. Bannister, R., Battershill, C. & De Nys, R. Suspended sediment grain size and mineralogy across the continental shelf of the Great Barrier Reef: impacts on the physiology of a coral reef sponge. *Cont. Shelf Res.* **32**, 86-95 (2012).

5. Muzuka, A. N., Dubi, A. M., Muhando, C. A. & Shaghude, Y. W. Impact of hydrographic parameters and seasonal variation in sediment fluxes on coral status at Chumbe and Bawe reefs, Zanzibar, Tanzania. *Estuar. Coast. Shelf S.* **89**, 137-144 (2010).

6. Mallela, J., Parkinson, R. & Day, O. An assessment of coral reefs in Tobago. *Caribb. J. Sci.* **46**, 83-87 (2010).

7. Golbuu, Y., Van Woesik, R., Richmond, R. H., Harrison, P. & Fabricius, K. E. River discharge reduces reef coral diversity in Palau. *Mar. Pollut. Bull.* **62**, 824-831 (2011).

8. Knapp, I. S. et al. Restriction of sponges to an atoll lagoon as a result of reduced environmental quality. *Mar. Pollut. Bull.* **66**, 209-220 (2013).

9. De'ath, G. & Fabricius, K. Water quality of the Great Barrier Reef: distributions, effects on reef biota and trigger values for the protection of ecosystem health. *Great Barrier Reef Marine Park Authority* **89**, 11 (2008).

10. Blomqvist, S. & Hakanson, L. A review on sediment traps in aquatic environments. *Arch. Hydrobiol.* **91**, 101-132 (1981).
